# ODoSE: A Webserver for Genome-Wide Calculation of Adaptive Divergence in Prokaryotes

**DOI:** 10.1371/journal.pone.0062447

**Published:** 2013-05-06

**Authors:** Michiel Vos, Tim A. H. te Beek, Marc A. van Driel, Martijn A. Huynen, Adam Eyre-Walker, Mark W. J. van Passel

**Affiliations:** 1 European Centre for Environment and Human Health, The University of Exeter Medical School, University of Exeter, Truro, United Kingdom; 2 Netherlands Bioinformatics Centre, Nijmegen, The Netherlands; 3 Centre for Molecular and Biomolecular Informatics, Radboud University Nijmegen Medical Centre, Nijmegen, The Netherlands; 4 Centre for the Study of Evolution, School of Life Sciences, University of Sussex, Brighton, United Kingdom; 5 Systems and Synthetic Biology, Wageningen University, Wageningen, The Netherlands; Université Claude Bernard - Lyon 1, France

## Abstract

Quantifying patterns of adaptive divergence between taxa is a major goal in the comparative and evolutionary study of prokaryote genomes. When applied appropriately, the McDonald-Kreitman (MK) test is a powerful test of selection based on the relative frequency of non-synonymous and synonymous substitutions between species compared to non-synonymous and synonymous polymorphisms within species. The webserver ODoSE (Ortholog Direction of Selection Engine) allows the calculation of a novel extension of the MK test, the Direction of Selection (DoS) statistic, as well as the calculation of a weighted-average Neutrality Index (NI) statistic for the entire core genome, allowing for systematic analysis of the evolutionary forces shaping core genome divergence in prokaryotes. ODoSE is hosted in a Galaxy environment, which makes it easy to use and amenable to customization and is freely available at www.odose.nl.

## Introduction

The immense genomic diversity of bacteria and archaea is rapidly being uncovered by next-generation sequencing methods. Much attention in comparative genomics studies is given to differences in gene content mediated by lateral gene transfer, gene duplication and gene loss, as related strains can differ markedly in gene content [1]. The accessory genome clearly is of profound importance to the physiology and ecology of strains and species. However, it has become increasingly clear that bacterial core genes conserved between species play a major role in niche adaptation as well [2]–[7]. The McDonald-Kreitman (MK) test is a powerful test of selection [8]–[10] comparing patterns of non-synonymous and synonymous substitutions within a species to those separating this species from an outgroup species. In the first large-scale application of the MK test to prokaryotes, it was estimated that at least 50% of amino acid changes fixed in *Escherichia coli* and *Salmonella enterica* core genes were due to adaptation [9], demonstrating that prokaryote sequence evolution can in large part be shaped by natural selection.

The MK test is based on the premise that under neutral evolution, the ratio of synonymous and nonsynonymous substitutions within a species is the same as that between this species and an outgroup species. If a species has diverged due to positive selection (having changed its phenotype), an excess of nonsynonymous changes is expected between species relative to that within species. This is because adaptive mutations are fixed relatively rapidly and so contribute little to intra-specific polymorphisms but do contribute to between-species divergence. In contrast, when deleterious mutations segregate within a species because of inefficient purifying selection, nonsynonymous polymorphisms are overrepresented and adaptive divergence is underestimated. The standard summary statistic of the MK test is the Neutrality Index NI [11]:
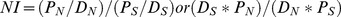
(1)


Where P_N_ and P_S_ are non-synonymous and synonymous polymorphisms and D_N_ and D_S_ are non-synonymous and synonymous fixed differences between species. With NI = 1, there is no difference in the pattern between non-synonymous and synonymous substitutions and species diverge neutrally. With NI<1, fixed differences between species are more often due to non-synonymous differences than expected and divergence is assumed to be selected for (positive selection). With NI>1, fixed differences between species are less often due to non-synonymous divergence than expected. This is caused by selection against protein-changes (negative selection), with divergence primarily being driven by neutral fixation of synonymous substitutions (drift). Statistical deviation from NI = 1 can be assessed using a 2×2 contingency table and a Chi squared test. In some studies the inverse of the Neutrality Index [11], the Fixation Index, is used (e.g. [12]).

Because the MK test statistic NI is based on a ratio of two ratios, it cannot be calculated for genes where D_N_ or P_S_ is 0. This thus results in discarding many genes from datasets (e.g. [13]). To counteract this problem, Stoletzki and Eyre-Walker [14] have proposed an alternative statistic, termed the Direction of Selection (DoS):
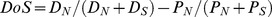
(2)


DoS can be calculated for all genes except those where both P_S_ and P_N_ and/or both D_N_ and D_S_ are zero. The significance of DoS can be assessed in the same way as NI.

It is useful to be able to quantify adaptive divergence for all orthologs shared between two taxa (the ‘core genome’). Summing polymorphism and divergence for all orthologs to calculate NI or averaging NI values for all individual orthologs however results in statistical bias [14]. To calculate an overall NI across genes Stoletzki and Eyre-Walker suggest using a variant of the Haenzsel-Mantel method for combining contingency tables. A novel weighted-average of the NI statistic for all shared genes, NI_TG_ (named after Tarone and Greenland) performs well regardless of heterogeneity of NI across genes and comes with a 95% Confidence Interval [14]:
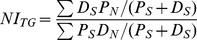
(3)


In order to provide a user-friendly method to apply the MK test of selection to entire bacterial core genomes, we have developed a web service with a graphical user interface called ODoSE (Ortholog Direction of Selection Engine). The ODoSE pipeline enables researchers to select prokaryote genomes of interest from the NCBI database and/or upload their own genome data, after which the DoS statistic is calculated for every individual single-copy ortholog (SICO) and the NI_TG_ statistic is calculated for all SICOs combined, allowing for the genome-wide characterization of adaptive divergence.

## Results

**Figure 1:**
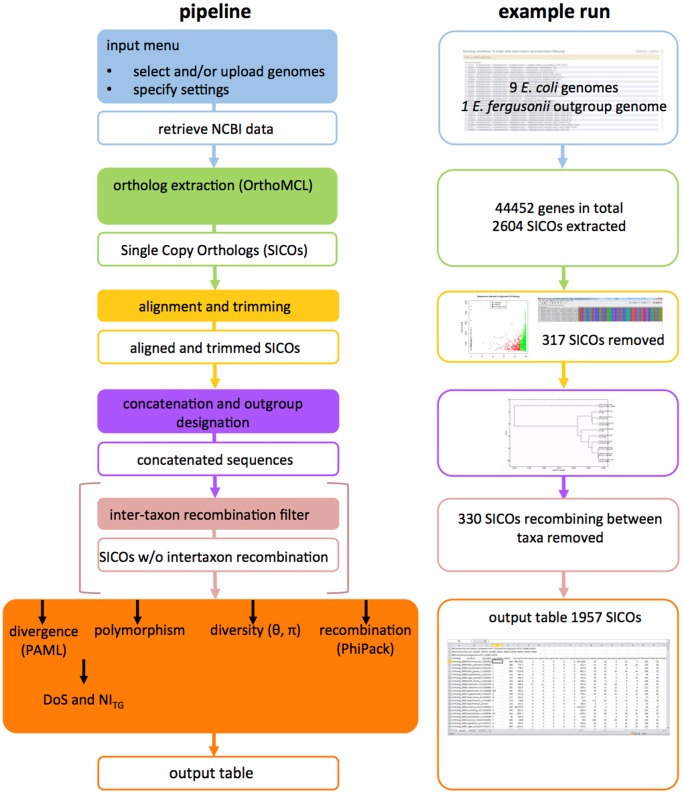
Schematic representation of the ODoSE workflow.

The ODoSE workflow is implemented in the Galaxy framework [15], which is supported by a large and active community, does not require programming experience or command line instructions and makes it easy to share results. Importantly, users have the possibility to customize the default workflow as they see fit. A brief overview of the pipeline is given below and in Figure 1. Log files are provided with each step in the analysis to summarize data and list any potential errors. A more extensive manual as well as two example runs are hosted on the ODoSE website www.odose.nl.

### Input

Genomes can be selected from all prokaryote genome projects deposited in NCBI RefSeq and the EMBL Nucleotide Sequence Database. All genomes are available through a daily updated mirror (mrs.cmbi.ru.nl) to guarantee access. User-generated genome data in the FASTA nucleotide coding region format (.ffn) can be uploaded in combination with GenBank data or can be analysed independently. Both downloaded DNA sequences and translated protein sequences can be downloaded in a zip file for each selected genome.

### Extraction

A protein-level reciprocal BLAST to identify all orthologous sequences is performed by OrthoMCL [16]. Users can specify a minimal protein length for analysis (default is set to 30 amino acids) and the e-value for the reciprocal BLAST (default 10^−5^). A table is produced listing the distribution of all genes in all selected genomes. A zip file containing all SICO DNA sequences is provided; the input menu also gives the option to download zip files containing multiple copy orthologs or orthologs that occur in a subset of genomes only.

### Alignment and Trimming

Each extracted Single Copy Orthologous (SICO) gene is aligned using MUSCLE [17], back translated and trimmed to equal length. Orthologs that do not match user-defined alignment quality control parameters (% alignment overlap and indel length) are excluded. Zip files are provided containing aligned-, aligned and trimmed- and low quality SICOs. A scatterplot summarizes the alignment and trimming statistics.

### Concatenation and Outgroup Designation

SICOs are concatenated for every selected genome and a UPGMA tree is constructed for these concatemers using dnadist and neighbour from the PHYLIP 3.69 package (http://evolution.genetics.washington.edu/phylip.html). The genome used as the outgroup in the MK analysis is automatically assigned on the basis of the first split in this tree. For some of the individual SICOs, inter-taxon recombination events will have changed the outgroup position, prohibiting MK test analyses. Therefore, the option is given to create UPGMA trees for each individual SICO to filter for congruency with the concatemer UPGMA tree in order to exclude such recombinants. Zip files containing concatemers, individual SICO alignments and SICOs listed per genome are provided. A PDF of the concatemer UPGMA tree is provided for visual reference.

### Population Genetic Calculations

The codeml program in the PAML package [18], is used to calculate synonymous and non-synonymous divergence from the outgroup sequence. The site frequency spectrum and nucleotide diversity (π and θ) are tabulated using custom scripts. Custom scripts are used to calculate the DoS statistic for every individual SICO and the NI_TG_ statistic with associated confidence interval for the concatemer [14]. The package PHIPACK [19] is used to perform three tests of homologous recombination. A final output table summarizes all results. When the outgroup consists of multiple strains and the test can be performed for both taxa, a second output table is produced.

## Discussion

A lack of software applications as well as statistical difficulties with the MK test have prevented it to be commonly used on a genome-wide scale [9], [20], [21]. The ODoSE pipeline offers an easy-to-use workflow to perform two new extensions of the MK test: to automatically quantify the impact of natural selection on every single gene shared by a taxonomic group of interest as well as all genes combined, allowing for systematic analysis of the evolutionary forces shaping core genome divergence. The use of the pipeline is not limited to the MK test but permits a wider range of population genomic analysis. For instance, the concatenated SICO sequences can be used to generate high-resolution phylogenetic trees, the distribution of all genes in all genomes is tabulated enabling pan genome analyses and zip files are provided with core and accessory genes (per genome and per gene) for downstream analyses. Finally, for more sophisticated analyses, the distribution of polymorphisms (the Site Frequency Spectrum) can be used as input for the program DFE-alpha, which uses a maximum-likelihood method to calculate the proportion of adaptive substitutions [22].
